# Unraveling the Cause of Hemolytic Anemia in the Emergency Department: A Destructive Path

**DOI:** 10.7759/cureus.115586

**Published:** 2026-09-01

**Authors:** Morgan Walker, Chris W Baugh

**Affiliations:** 1 Department of Emergency Medicine, Brigham and Women's Hospital, Boston, USA

**Keywords:** anemia, diffuse large cell lymphoma, hematology, hemolytic anemia, hypotension, infectious disease, lymphoproliferative malignancy, lymphoproliferative process, malignancy, shortness of breath

## Abstract

Hemolytic anemia is a rare but potentially life-threatening condition in the emergency department (ED) that requires timely recognition and early intervention. Hemolytic anemia results from either the intravascular or extravascular breakdown of red blood cells. The most common causes of secondary hemolytic anemia include lymphoproliferative disorders, autoimmune disorders, and infections. This case will demonstrate how an urgent diagnosis of hemolytic anemia can lead to the underlying cause: diffuse large B-cell lymphoma. This case illustrates how laboratory evaluation, early subspecialty involvement, and maintenance of a broad differential are integral in a patient whose initial presentation is indistinguishable from other causes of hemolytic crisis.

A 61-year-old man presented to the ED complaining of worsening dyspnea on exertion and fatigue for two weeks. He arrived at the ED with stable vital signs. His vitals were as follows: blood pressure 118/66 mmHg, heart rate 72 beats per minute (bpm), respiratory rate 16 breaths per minute, temperature 97.8°F, and oxygen saturation 100% on room air. Laboratory evaluation revealed a hemoglobin of 7.6 g/dL, hematocrit of 21.4%, lactate dehydrogenase at 416 U/L, indirect hyperbilirubinemia of 2.8 mg/dL, and haptoglobin at <5 mg/dL. The patient was admitted to the hospital where he experienced multiple episodes of acute hemodynamic decompensation and underwent further diagnostic evaluation, ultimately resulting in a diagnosis of diffuse large B-cell lymphoma.

This case highlights how hemolytic anemia can mimic more common presentations such as acute blood loss, viral hepatitis, sepsis, autoimmune disorders, or malignancies. Awareness of its hallmark laboratory pattern and initiation of therapy are critical for optimal patient outcomes in the ED setting. The laboratory findings and clinical presentation of hemolytic anemia require early detection, as they may reflect life-threatening conditions such as autoimmune hemolytic anemia, glucose-6-phosphate dehydrogenase deficiency, or thrombotic microangiopathies including thrombotic thrombocytopenic purpura and hemolytic uremic syndrome. This case is notable for three distinct features: the diagnostic workup spanned two continents and was complicated by incomplete prior investigations; bilateral deep vein thromboses provided an early but underappreciated clue to an underlying hypercoagulable lymphoproliferative process; and autoimmune hemolytic anemia preceded and ultimately led to the diagnosis of diffuse large B-cell lymphoma, highlighting a diagnostic sequence with significant implications for emergency physicians.

## Introduction

A 61-year-old man presented to the emergency department (ED) with worsening dyspnea on exertion and fatigue for two weeks. He was told he had hemolytic anemia at an outside hospital before presenting to our institution. Hemolytic anemia is a rare but potentially life-threatening condition and requires expeditious recognition for early intervention. Hemolytic anemia occurs when red blood cells are destroyed faster than the bone marrow can replace them, resulting in progressive anemia. This case examines autoimmune hemolytic anemia secondary to an underlying B-cell lymphoproliferative disorder, a clinically significant yet often underrecognized association in the ED [1-3].

Hemolytic anemia can be classified into two primary categories: intrinsic and extrinsic. Intrinsic causes arise from defects within the red blood cells themselves, such as hereditary spherocytosis or glucose-6-phosphate dehydrogenase deficiency. Extrinsic causes involve external factors that damage red blood cells, including autoimmune reactions, infections, or mechanical trauma from prosthetic heart valves. The pathophysiology of hemolytic anemia involves either intravascular hemolysis, where red blood cells are destroyed within the bloodstream, or extravascular hemolysis, primarily occurring in the spleen and liver. The clinical presentation can vary from mild fatigue and pallor to severe manifestations like jaundice, dark urine, and splenomegaly, depending on the rate and extent of red blood cell destruction. Initiating the differential in the ED can dictate management and approach, which may range from supportive care and blood transfusions to immunosuppressive therapy or specific interventions targeting the underlying etiology. This case explores the development of hemolytic anemia secondary to B-cell lymphoma, resulting either from antibodies produced by malignant B cells that directly target red blood cells or from more widespread immune dysregulation caused by the lymphoma [2,3].

## Case presentation

A 61-year-old man with a history of well-differentiated squamous cell carcinoma of the right elbow presented to the ED with dyspnea on exertion, congestion, intermittent fevers, and severe fatigue for one week. Upon symptom onset, he presented to urgent care and was diagnosed with a sinus infection and completed a course of amoxicillin/clavulanic acid. Given his positive response to antibiotics, he traveled to Portugal for vacation, where he found himself deteriorating within two days of arriving with profound weakness, lightheadedness, and dyspnea on exertion. He presented to a local ED in Lisbon, where he was admitted for two days. He reports he was diagnosed with hemolytic anemia and given two red blood cell transfusions and three days of an unknown dose of prednisone. The patient had a hemoglobin of 5.9 g/dL, a white blood cell count of 15 K/µL, and a normal platelet count. He got two units of packed red blood cells and had an appropriate bump in his hemoglobin to 6.7 g/dL. His hospitalization was brief while abroad given that he wished to fly home to undergo a workup in the United States. He was dosed with steroids prior to any knowledge that he might need a biopsy in the near future.

Upon arrival to our institution, he was found to have a pale appearance, 1+ pitting edema in the bilateral lower extremities, and a palpable spleen tip below the costal margin; these symptoms were not previously reported in Lisbon. Preliminary lab results (Table 1) revealed a white blood cell count of 17.63 K/µL, a hemoglobin of 7.6 g/dL, a hematocrit of 21.4%, a platelet count of 373×10⁹/L, a reticulocyte count of 8.7%, a total bilirubin of 2.8 mg/dL, a direct bilirubin of 0.9 mg/dL, a lactate dehydrogenase of 416 U/L, a fibrinogen level of 287 mg/dL, and a haptoglobin level of <5 mg/dL. White blood cell differentiation revealed an absolute neutrophil count of 15.09 K/µL. His prothrombin time was 13.5 seconds with an international normalized ratio of 1.2. For comparison, the patient had normal lab work approximately 10 months prior at his primary care physician's office. The patient had a negative heterophile (Monospot) antibody test. Inflammatory markers included a normal sedimentation rate of 1 mm/h and an elevated C-reactive protein level of 28.7 mg/L.

**Table 1:** Laboratory results

Laboratory test	Result	Units	Reference range
White blood cell count (WBC)	17.6	K/µL	4.0-11.0
Absolute neutrophil count (ANC)	15.0	K/µL	1.5-8.0
Hemoglobin	7.6	g/dL	12.0-16.0 (female); 13.5-17.5 (male)
Hematocrit	21.4	%	36-46 (female); 41-53 (male)
Platelet count	373	×10⁹/L	150-400
Reticulocyte count	8.7	%	0.5-2.5
Total bilirubin	2.8	mg/dL	0.2-1.2
Direct bilirubin	0.9	mg/dL	0.0-0.3
Lactate dehydrogenase (LDH)	416	U/L	140-280
Fibrinogen	287	mg/dL	200-400
Haptoglobin	<5	mg/dL	30-200
Erythrocyte sedimentation rate (ESR)	1	mm/h	0-20
C-reactive protein (CRP)	28.7	mg/L	<8
Prothrombin time (PT)	13.5	seconds	11-13.5
International normalized ratio (INR)	1.2	N/A	0.8-1.1

His chest radiograph did not demonstrate pneumonia; however, possible trace bilateral pleural effusions were present (Figure 1).

**Figure 1:**
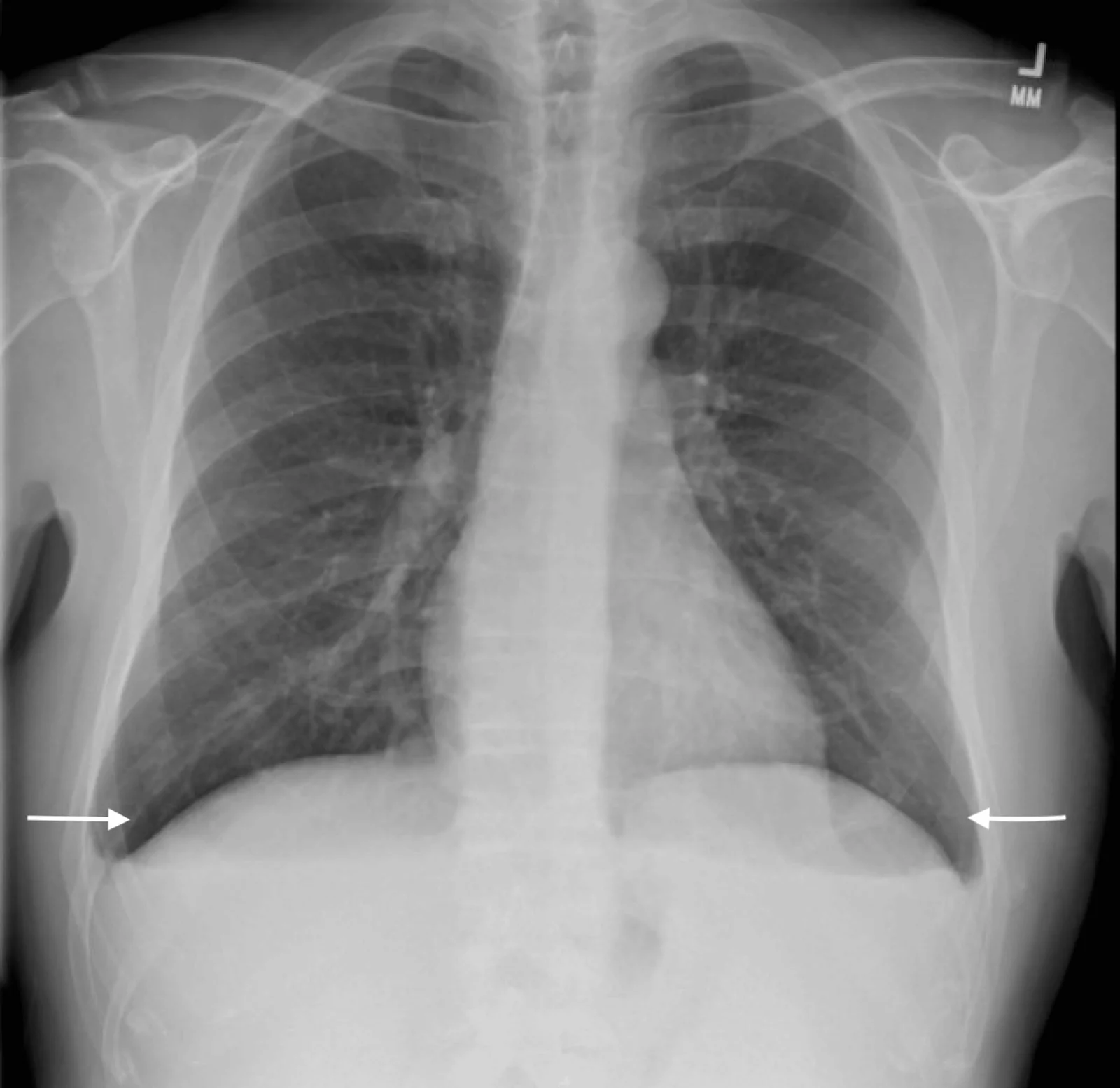
Chest X-ray Chest radiograph did not demonstrate pneumonia; however, possible trace bilateral pleural effusions were present. The arrows depict trace pleural effusions. There is no overt consolidation or tracheal deviation, reassuring against pneumonia or tension pneumothorax, respectively.

A limited point-of-care ultrasound at bedside upon arrival was negative for obvious pericardial effusion and valvular vegetations and did not demonstrate any evidence of right heart strain. Abdominal ultrasound revealed splenomegaly with numerous hypoechoic masses, which showed vascularity, the largest 5.1×4.2×3.1 cm, and liver cysts and hyperechoic lesions that could represent hemangiomas (Figures 2-3).

**Figure 2:**
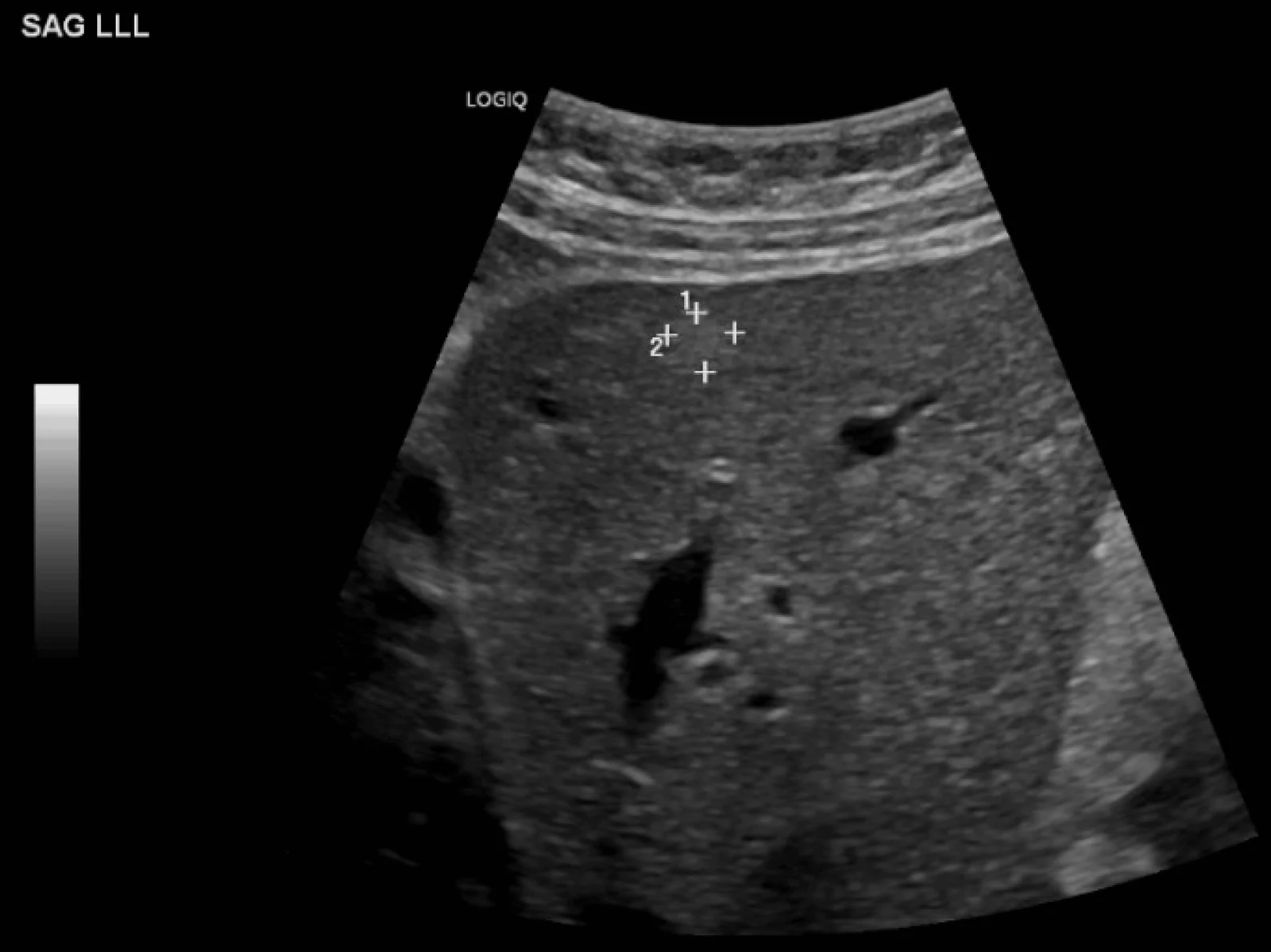
Abdominal ultrasound This is an ultrasound snapshot of the liver in the right upper quadrant of the abdomen. The hyperechoic lesion in the liver is denoted by 1 and 2 with white outline and depicts one angle of a liver lesion or hemangioma.

**Figure 3:**
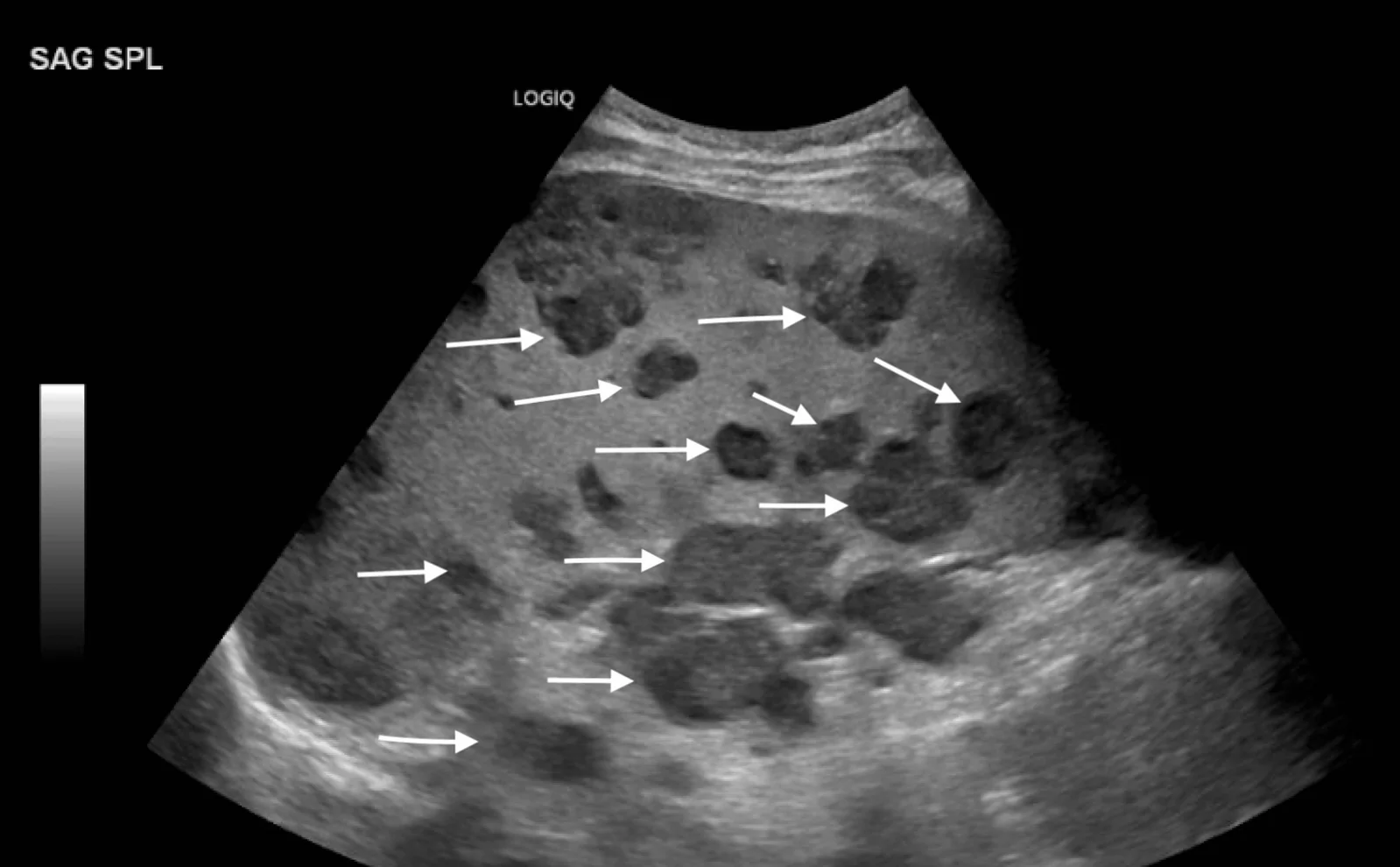
Abdominal ultrasound This is an ultrasound snapshot of the spleen. It shows the spleen which is very enlarged with numerous hypoechoic masses. The many white arrows indicate numerous hypoechoic masses within the spleen.

Computed tomography (CT) scan of the chest demonstrated enlarged left supraclavicular lymph nodes, small bilateral pleural effusions, and multiple small pulmonary nodules. The CT scan of the abdomen/pelvis demonstrated marked splenomegaly with innumerable hypodense splenic lesions and multiple enlarged upper abdominal/retroperitoneal lymph nodes (Figure 4).

**Figure 4:**
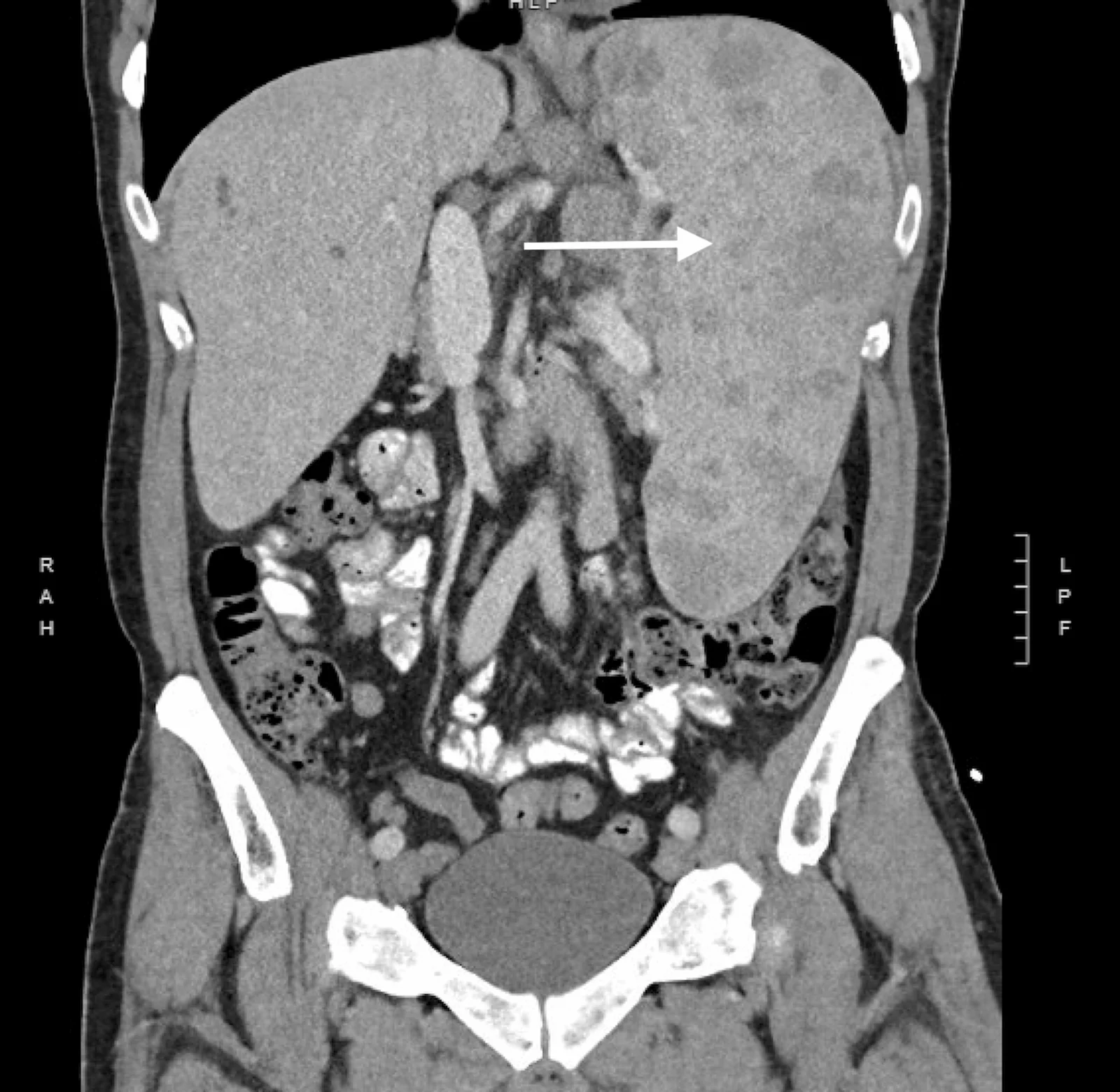
Computed tomography As seen in the computed tomography scan, there is marked splenomegaly with innumerable hypodense splenic lesions. The arrow indicates the spleen which is enlarged. There are multiple hypodense lesions in the spleen that are better depicted in Figure 3.

Pulmonary embolism was considered in the ED given the constellation of recent long flights and dyspnea on exertion. The Wells criteria was calculated to assess the patient's pretest probability of pulmonary embolism. Based on the absence of clinical risk factors included in the scoring system, the patient had a Wells score of 0, corresponding to a low pretest probability of pulmonary embolism.** **A point-of-care bedside ultrasound demonstrated no evidence of right heart strain, also reassuring against a hemodynamically significant pulmonary embolism. The initial CT scan of the chest was not protocolled as a pulmonary angiogram study. However, while the patient was admitted, he had a chest CT pulmonary angiogram that was negative for pulmonary embolism.

There were multiple lucent osseous lesions, likely osseous involvement by the suspected lymphoproliferative process. Additional findings included indeterminate hepatic lesions that may correspond to the echogenic lesions on ultrasound, with a differential diagnosis including hemangiomas. Ultrasound of the bilateral lower extremities revealed deep vein thrombosis of the right and left gastrocnemius veins, which appeared to be acute (Figures 5-6). The occurrence of hyperacute thrombotic events prompted consideration of an underlying lymphoproliferative disorder. The clinical presentation raised concern for an associated malignancy-induced hypercoagulable state.

**Figure 5:**
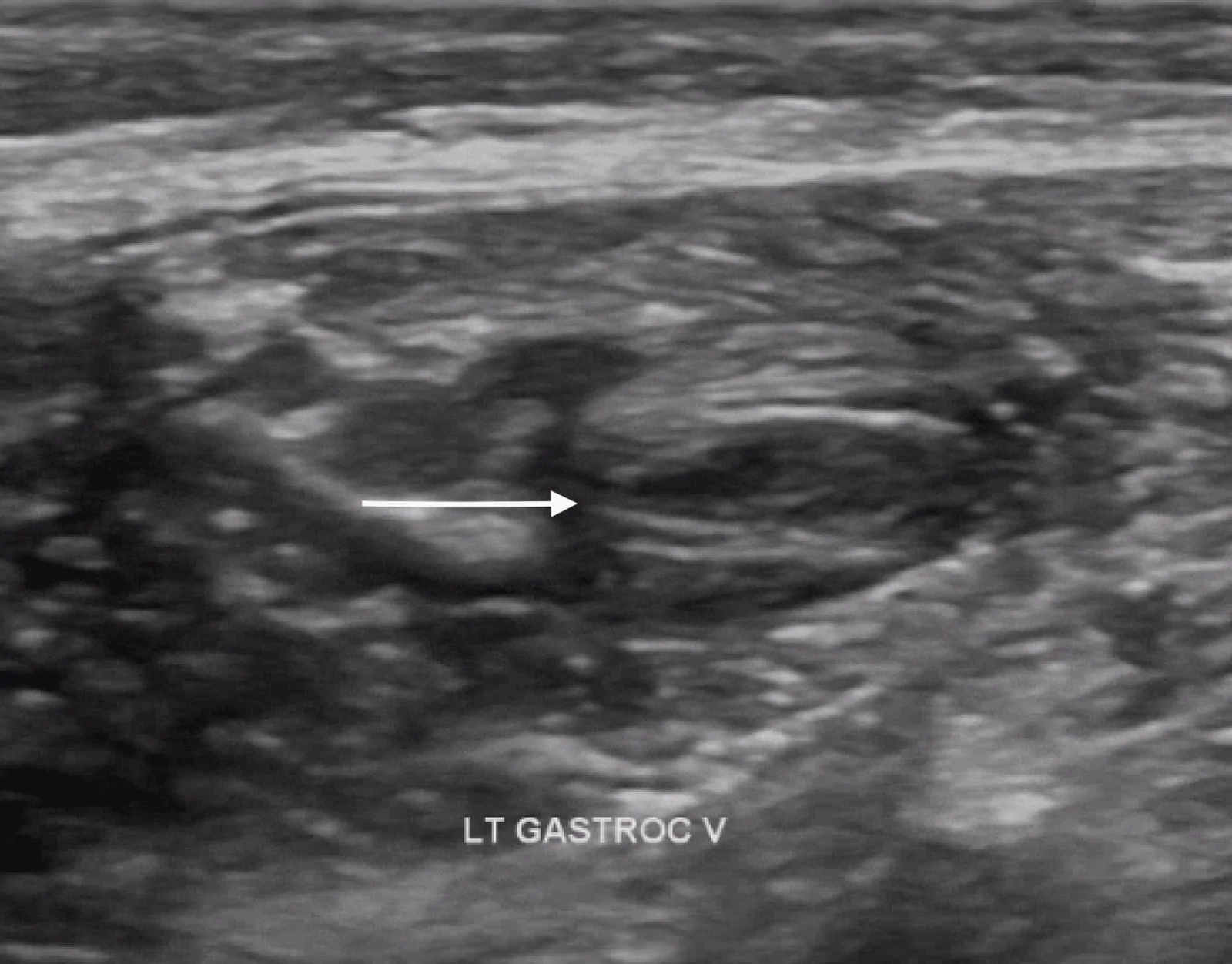
Deep vein thrombus ultrasound of the left leg Evidence of a deep vein thrombus in the left gastrocnemius vein. The white arrow depicts an incompressible vein (thrombus). Although it is difficult to visualize the compressibility in a still image, the vein is incompressible with clot burden within the walls of the vein.

**Figure 6:**
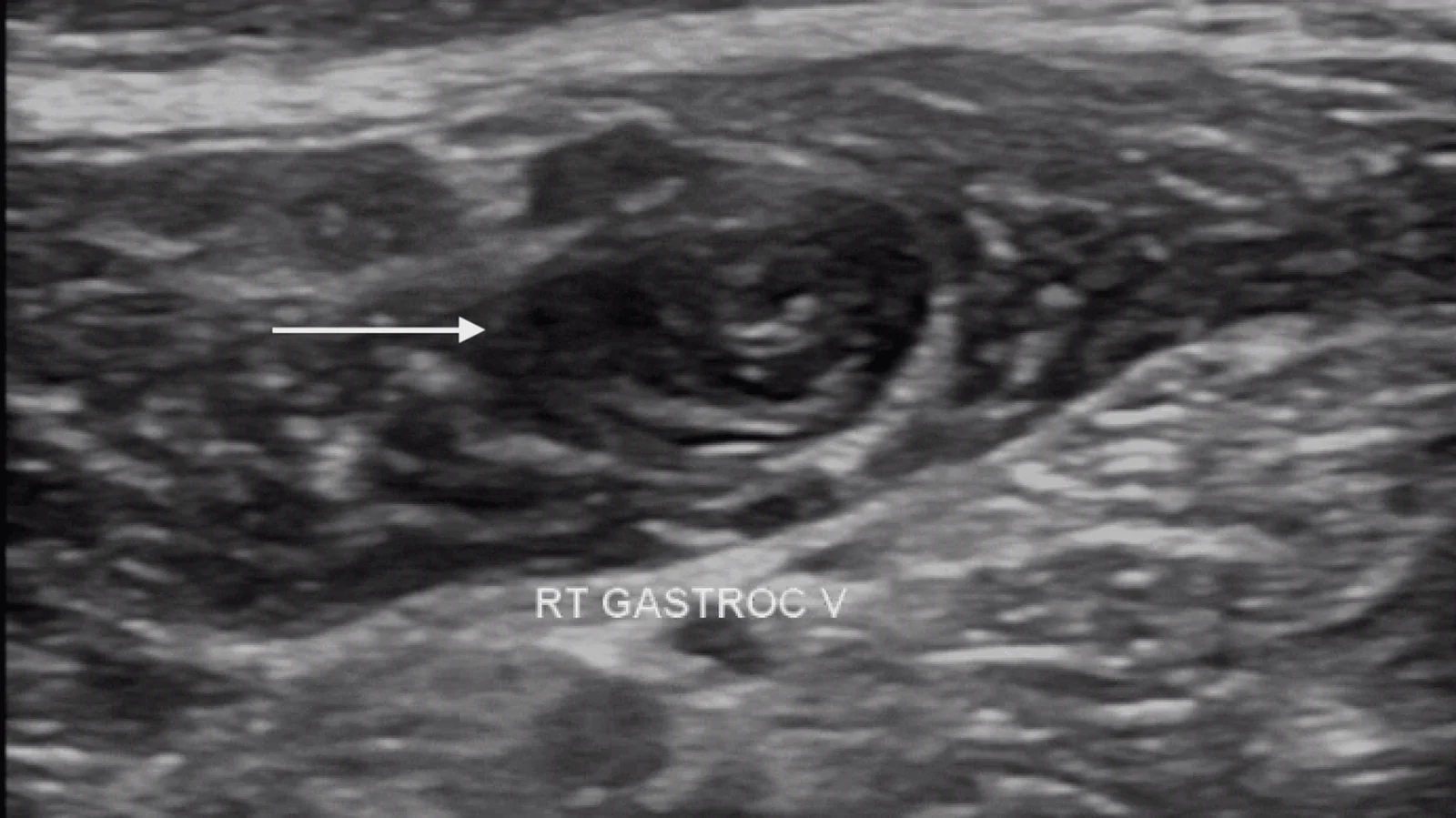
Deep vein thrombus ultrasound of the right leg Evidence of a deep vein thrombus in the right gastrocnemius vein. The white arrow indicates an incompressible vein (thrombus).

Initially, the patient's hemolytic anemia was extremely concerning for a malignant process; however, the hospital team was also considering autoimmune or infectious processes. The patient was admitted to the general medicine team given the broad differential. His presentation was inconsistent with thrombotic thrombocytopenic purpura, given a normal platelet count. His constitutional symptoms, including weight loss, night sweats, and fatigue, were more consistent with malignancy. The patient's lab work demonstrated that only one cell line was decreased, which could challenge the suspicion of a marrow process. Additionally, he did not endorse any sick contacts, exposure to ticks/insects, or additional travel prior to Portugal. While admitted, a broad differential was being considered as the patient had no other risk factors or explanations for his constitutional symptoms.

Additional studies were analyzed during inpatient care. This included negative blood cultures, a positive direct Coombs test, a positive IgG direct antiglobulin test, and a positive C3 direct antiglobulin test. The patient was originally found to have warm autoimmune hemolytic anemia that was later specified as mixed warm and cold autoimmune anemia, after both positive results for cold and warm autoimmune antibodies. Of note, the mainstay of treatment for warm/cold autoimmune hemolytic anemia is to treat the underlying cause which was suspected to be lymphoproliferative malignancy. A flow cytometry analysis of the peripheral blood specimen demonstrated a population of B cells that was positive for B-lymphoid markers CD19, CD20, and surface immunoglobulin kappa. The findings on the peripheral blood smear were not definitive for B-cell lymphoma (Figure 7). A cervical lymph node biopsy demonstrated histopathology consistent with diffuse large B-cell lymphoma confirming the suspected lymphoproliferative process.

**Figure 7:**
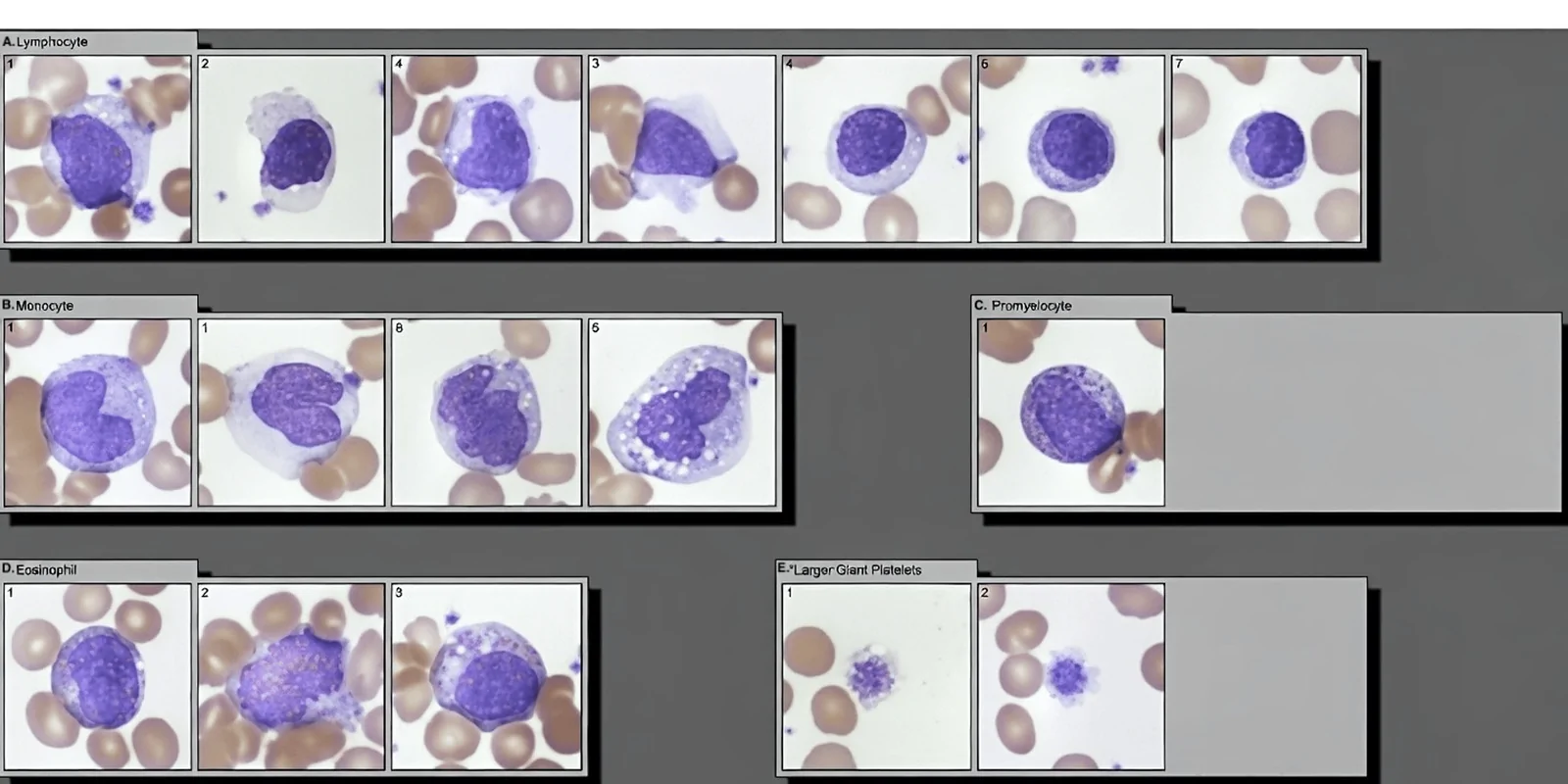
Peripheral smear Peripheral blood smear (Wright-Giemsa stain, 40× magnification) obtained at the time of presentation. Labeled elements include the following: (A) lymphocyte, (B) monocyte, (C) promyelocyte, (D) eosinophil, and (E) "larger" giant platelet components. The white cells are elevated in number and show neutrophil toxic granulation. No atypical lymphocytes are noted. There are no blasts or immature elements. Platelets are normal in number with normal morphology and granularity; some giant platelets are present.

One day after presenting to the hospital, while the patient was admitted, a rapid response was called secondary to the patient becoming significantly hypotensive to 84/46 mmHg and hypoxic with oxygen desaturations to the 80s, for which he required 3 L of oxygen via a nasal cannula. He was empirically provided antibiotics with cefepime and vancomycin for presumed sepsis as well as given intravenous fluids and never required intensive care unit level of care. Over the next three days, multiple rapid responses were initiated in response to recurrent episodes of hemodynamic instability, each of which was transient.

On the third day of the patient's admission, the hematology team evaluated the patient. They noted that his lab work showed evidence for warm autoimmune hemolytic anemia, massive splenomegaly, and diffuse lymphadenopathy. The constellation of clinical findings raised suspicion for a lymphoproliferative disorder complicated by warm autoimmune hemolytic anemia. They recommended a core tissue biopsy of the supraclavicular lymph nodes and many of his intra-abdominal lymph nodes. After reviewing serial daily lab work, the hematology team noted that he was hemolyzing at an acceptable rate. It was reasonable to defer additional corticosteroids to preserve diagnostic integrity, given the trend of his hemoglobin and hematocrit, all while keeping in mind the steroids he already received in Portugal. His lab trend for hemoglobin and hematocrit did not show a profound downtrend that required urgent steroids, despite hemodynamic instability at times. The recommended treatment was to transfuse to a hemoglobin level of >7 g/dL. The patient received several units of packed red blood cells during his stay inpatient. As a temporizing measure, 1 g/kg of intravenous immunoglobulin was administered. Once a diagnostic tissue biopsy confirmed diffuse large B-cell lymphoma, the hematology team guided prednisone therapy at 1 mg/kg PO daily. Rituximab was administered once the diagnostic biopsy confirmed the diagnosis.

Given that various infectious syndromes could account for the patient's constellation of symptoms, the infectious disease team recommended testing for cytomegalovirus, Epstein-Barr virus, mycoplasma, *Babesia*, malaria, human immunodeficiency virus, hepatitis, and a parasite smear, as these are certain infections that could cause autoimmune hemolytic anemia, including immune-mediated or hemophagocytic lymphohistiocytosis-like syndromes. All of the infectious disease testing was negative. The infectious disease team recommended continuing vancomycin, cefepime, and metronidazole while the infectious sources were being ruled out.

Following the biopsy confirmation of diffuse large B-cell lymphoma, the patient was started on prednisone at 1 mg/kg/day and rituximab. Hemoglobin stabilized above the transfusion level with a declining retic count consistent with improved hemolysis. The patient was discharged after several weeks in stable condition.

## Discussion

Autoimmune hemolytic anemia can encompass a wide variety of differentials. If gone unrecognized, hemolytic anemia can have the potential for rapid decompensation and life-threatening complications. Autoimmune hemolytic anemia may present with generic, nonspecific signs such as jaundice, dark urine, hypotension, or acute renal injury. Significant delays in diagnosis could result in multi-organ dysfunction [4-6]. Many complex cases of autoimmune hemolytic anemia yield a broad differential upon presentation to the ED. Viruses such as parvovirus B19 and hepatitis C show an increased incidence of autoimmune hemolytic anemia. Additional diagnoses, such as infectious mononucleosis and *Mycoplasma pneumoniae* infection, are associated with cold agglutinin autoimmune hemolytic anemia, which occurs in approximately 3% of patients. Lymphoproliferative disorders are suspected when autoimmune hemolytic anemia does not respond to first-line steroids. Chronic lymphocytic leukemia is a subtype of lymphoproliferative disorders that has a 5-10% risk of developing autoimmune hemolytic anemia [5,6].

Thrombotic thrombocytopenic purpura causes microangiopathic hemolysis, which carries a high mortality rate unless plasma exchange, corticosteroids, and adjunctive therapies are initiated without delay [7,8]. In patients with enzymatic defects, such as glucose-6-phosphate dehydrogenase deficiency, exposure to oxidant stressors (e.g., certain medications, infections, or foods) can precipitate hemolytic crises. As clinicians, we must recognize triggers and anticipate hemolysis, even when laboratory values do not overtly suggest acute decompensation [9-11].

Given the spectrum of causes, overlapping presentations, and high stakes, ED clinicians must distinguish hemolytic processes using laboratory evaluation (e.g., complete blood count, blood smear, haptoglobin, lactate dehydrogenase, direct antiglobulin testing, and ADAMTS13 when indicated). Autoimmune hemolytic anemia may be the first manifestation of an underlying lymphoproliferative disorder and can precede the diagnosis of malignancy itself. Failure to identify this connection may delay appropriate treatment, resulting in ongoing hemolysis, worsening anemia, and progression of the underlying hematologic process [12].

This case is distinguished by recurrent episodes of hemodynamic instability secondary to severe autoimmune hemolytic anemia, highlighting the potential severity of B-cell-driven hemolysis. Bilateral lower extremity deep vein thromboses provided an early, underappreciated clue to an underlying lymphoma-associated hypercoagulable state. Furthermore, the overlapping presentations of autoimmune hemolytic anemia, sepsis, and fever created a complex diagnostic challenge that required simultaneous multispecialty evaluation and ultimately facilitated the diagnosis of the underlying lymphoproliferative disorder. Clinicians in the ED must anticipate several acute potential complications of autoimmune hemolytic anemia, including acute kidney injury, neurologic involvement, and complications of microangiopathic processes. Massive intravascular hemolysis releases free hemoglobin and heme proteins, leading to hemoglobinuria and direct tubular injury. Acute kidney injury is common in hemolytic uremic syndrome, occurring in approximately 55-70% of patients, of which most recover renal function with supportive care. In microangiopathic hemolysis (e.g., thrombotic thrombocytopenic purpura and hemolytic uremic syndrome), there is also a risk of bleeding due to platelet destruction, thrombotic end-organ damage, and coagulopathic complications [12-14].

Neurologic complications such as seizures, altered mental status, and even posterior reversible encephalopathy syndrome may occur, particularly when hemolysis is severe or when associated with hypertensive crises. Early discernment of these potential complications while monitoring renal function, fluid status, mental status, and coagulation parameters is integral for improved outcomes [15,16].

Acute thrombotic events, such as pulmonary embolism, are typically related to active hemolysis and an increased risk of mortality. In cases where anemia is severe (hemoglobin <6 g/dL), combined with acute renal failure, infection, and immune thrombocytopenia, the mortality rate is about 30-57%. In a nationwide study of more than 2,500 patients with autoimmune hemolytic anemia, including both primary and secondary cases, Hansen et al. reported a one-year survival rate of 82.7% and a median survival of 9.8 years among patients with primary autoimmune hemolytic anemia. In comparison, patients with secondary autoimmune hemolytic anemia had a one-year survival rate of 69.1% and a median survival of 3.3 years. This study highlights the substantial burden associated with autoimmune hemolytic anemia when it occurs in conjunction with underlying lymphoproliferative malignancies [17]. Autoimmune hemolytic anemias that require intensive care unit-level care demand a multimodal approach for managing acute hemolysis. Interventions used in the intensive care unit can include, but are not limited to, preparing transfusions, steroid boluses, intravenous immunoglobulin, rituximab, erythropoietin, plasma exchange, and urgent splenectomy. These are often used in combination as a last resort for managing acute hemolysis [7].

It is important to note that the patient's prior hospitalization in Lisbon established the diagnosis of autoimmune hemolytic anemia and provided pre- and post-transfusion hemoglobin values, which helped direct the initial diagnostic evaluation at our institution. More broadly, this case highlights an important principle for emergency medicine providers: while a complete etiologic workup may not be achievable in the ED, timely recognition of autoimmune hemolytic anemia through characteristic laboratory findings, including low haptoglobin, elevated lactate dehydrogenase, indirect hyperbilirubinemia, and reticulocytosis, represents a critical contribution of emergency care and facilitates appropriate downstream evaluation and management.

## Conclusions

This case highlights the importance of keeping a broad differential diagnosis in patients with vague symptoms in the ED. Autoimmune hemolytic anemia is not an immediately obvious diagnosis in the ED, yet the condition can rapidly progress and be life-threatening. Autoimmune hemolytic anemia can be the initial and sole presentation of diffuse large B-cell lymphoma, a diagnosis with a substantially worse prognosis than primary autoimmune hemolytic anemia. Although uncommon, autoimmune hemolytic anemia may present with nonspecific features such as fever, hypotension, and imaging abnormalities, which could hinder a timely diagnosis. Immediate consultation with subspecialists, while patients are still in the ED, and initiation of appropriate therapy are critical to improving outcomes. Treatment options for autoimmune hemolytic anemia can include preparing transfusions, steroid boluses, intravenous immunoglobulin, rituximab, erythropoietin, plasma exchange, and urgent splenectomy.

Secondary autoimmune hemolytic anemia is associated with substantial mortality and significantly poorer outcomes compared with primary autoimmune hemolytic anemia. This underscores that emergency physician recognition of the condition, and the subsequent cascade of subspecialty evaluation it initiates, is not merely a diagnostic exercise, but a critical factor influencing long-term patient prognosis.

As an emergency medicine provider, early clinical suspicion of hemolytic anemia can prevent hemodynamic instability, worsening tissue hypoxia, and end-organ damage. Identifying the situation from an ED perspective may improve overall outcomes and survival.
